# Impact of High Pressure Processing on the In Vitro Bioaccessibility of Polyphenols in Sour Cherry (Prunus cerasus L.) Juice

**DOI:** 10.1021/acsomega.5c02380

**Published:** 2025-05-06

**Authors:** Gulay Ozkan, Senem Kamiloglu, Nurullah Demir, Esra Capanoglu

**Affiliations:** † Department of Food Engineering, Faculty of Chemical and Metallurgical Engineering, 52971 Istanbul Technical University, Maslak 34469, Istanbul, Türkiye; ‡ Department of Food Engineering, Faculty of Agriculture, Bursa Uludag University, Gorukle 16059, Bursa, Türkiye; § Science and Technology Application and Research Center (BITUAM), Bursa Uludag University, Gorukle 16059, Bursa, Türkiye; ∥ Department of Food Processing, Vocational School of Food, Agriculture and Livestock, 162312 Bingöl University, Bingöl 12000, Türkiye

## Abstract

As the demand for minimally processed food products with fresh-like characteristics continues to grow, nonthermal techniques are becoming increasingly popular. Fruit juices, due to their significant health benefits, are excellent candidates for nonthermal treatments aimed at preserving maximum nutritional value. Sour cherry (Prunus cerasus L.) juice (SCJ) is particularly rich in polyphenols, especially anthocyanins. This study comparatively assessed the impact of high-pressure processing (HPP) (300–400–500 MPa/5–10–20 min) on the content and in vitro bioaccessibility of total phenolics (TPC), flavonoids (TFC), monomeric anthocyanins (TMAC), antioxidant capacity (TAC), and individual polyphenolic compounds in SCJ. The findings revealed that both thermal pasteurization and HPP treatments significantly enhanced polyphenol bioaccessibility in SCJ compared to untreated samples. Thermal treatment resulted in the highest bioaccessibility levels for TMAC (82%), TAC (120%), and individual polyphenolic compounds (86%). HPP-treated samples exhibited greater TFC bioaccessibility (60–82%) than thermally pasteurized SCJ (51%), with samples processed at 500 MPa showing improved bioaccessibility across most phenolic fractions due to enhanced cell permeability and mass transfer. However, HPP generally reduced TAC bioaccessibility (60–78%) compared to the control (83%), except under high-pressure conditions (500 MPa for 20 min), highlighting the complex interplay between processing parameters and polyphenol stability.

## Graphical abstract

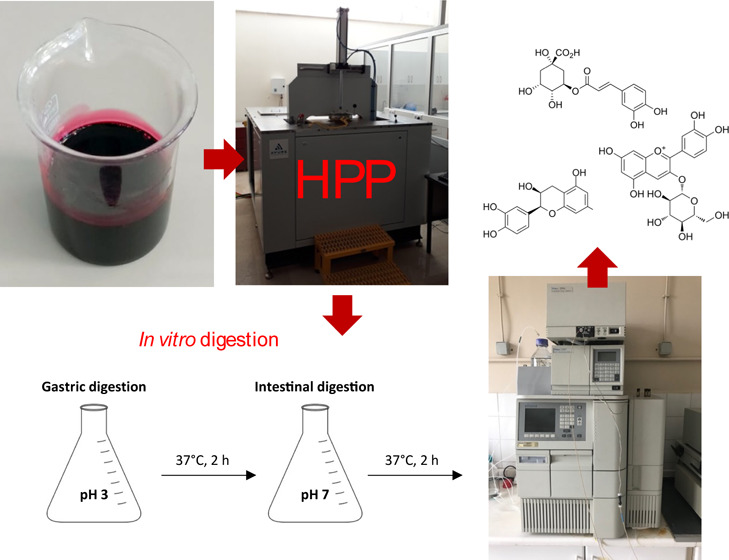

## 1 Introduction

As consumers increasingly demand minimally processed and nonchemically preserved food products with fresh-like characteristics, the application of nonthermal techniques has been gaining popularity.^1^ While widely used thermal treatments for preserving food products have certain advantages, they also come with significant disadvantages such as altering the quality characteristics and reducing the level of bioactive compounds due to the high temperatures involved.^2^ Given that extending shelf life and ensuring food safety are primary concerns in the food industry, there is a necessity for nonthermal technologies that can fulfill these requirements.^3^ One promising technology which has gained attention recently as a potential alternative to thermal processing is high-pressure processing (HPP). The use of HPP as a nonthermal method for food processing has shown significant progress in the past years.^4^

According to the latest information from the FAO, worldwide sour cherry production has reached around 1.6 million tons. Türkiye ranks fourth among the leading global producers, with an annual production of 211,291 tons.^5^ Sour cherries are typically consumed by being processed into sour cherry juice (SCJ), a process that involves thermal treatments such as pasteurization.^6^ Studies have demonstrated that subjecting sour cherries to heat treatment may lead to reduction in polyphenol levels, particularly anthocyanins.^7,8^ On the other hand, HPP could be an effective alternative to overcome this issue. HPP is one of the most advanced and widely adopted nonthermal preservation technologies in the food industry. Since 1990, HPP has been extensively applied worldwide, both in commercial and in laboratory settings, for preserving various foods.^9^ The use of HPP for the preservation of foods has also been approved by FDA and has been successfully applied to many food products including fruit juices.^10^ So far, a number of studies have been conducted on the effect of HPP treatment on the polyphenol content of fruit juices, including orange,^11^ apple^12^ and grape^13^ juices.

The polyphenols found in sour cherries provide functional properties that aid in preventing various diseases due to their potent antioxidant, antidiabetic, antiobesity, antimutagenic, and anticarcinogenic properties.^14^ Bioaccessibility of polyphenols within the gastrointestinal tract is widely acknowledged as essential for their health-promoting effects.^15^ Researchers have developed in vitro digestion models to explore the bioaccessibility of polyphenols from diverse food sources. These models offer benefits such as rapidity, affordability, and absence of ethical constraints linked with human studies.^16^ In vitro digestion models have already been used to investigate changes in the bioaccessibility of polyphenols in fruit juices subjected to HPP.^17–19^

The existing literature on the effect of HPP on sour cherry polyphenols is quite limited,^20^ and to the best of our knowledge, no previous studies have investigated the effects of HPP on the in vitro bioaccessibility of polyphenols from SCJ. Given that the aim of this study was to assess the impact of HPP treatment at varying pressures (300–400–500 MPa) and durations (5–10–20 min) on the content and in vitro bioaccessibility of total phenolics, flavonoids, anthocyanins, antioxidant capacity, and individual polyphenolic compounds in SCJ. In addition, thermal pasteurization was also applied to compare the results with those obtained from HPP treatment.

## 2 Materials and Methods

### 2.1 Materials and Chemicals

SCJ concentrates were obtained from Erkon Konsantre Inc. (Konya, Türkiye), frozen in aseptic bottles, and stored at −20 °C until use. The bottles were aseptically opened, and the samples were prepared by diluting the concentrates to 13.5 °Brix (degrees Brix). Subsequently, the samples underwent HPP treatment.

All chemicals and reagents used in this study were of analytical or HPLC grade and were obtained from Sigma-Aldrich (Steinheim, Germany), unless specified otherwise. The quantification of major polyphenolic compounds was performed using the following standards: epicatechin (≥98%), chlorogenic acid (≥95%), cyanidin 3-*O*-glucoside (≥96%; Extrasynthese, Genay, France), quercetin 3-*O*-glucoside (≥98%), rutin (≥94.0%), and kaempferol (≥97.0%).

### 2.2 High Pressure Processing of SCJ

HPP processing of SCJ samples was conducted using high-pressure equipment (Avure Technologies Inc., Columbus, OH, USA), with water serving as the pressure transmitting medium. The juice samples were subjected to pressures of 300, 400, and 500 MPa for durations of 5, 10, and 20 min at ambient temperature. Pressure increases and release times were excluded from the treatment durations for the samples reported in this study. Three pouches per treatment, each containing 100 mL of sample, were introduced into the pressure vessel. All treatments were conducted in triplicate. After HPP treatment, the pouches were stored at 4 °C for analysis within 24 h. Subsequently, all samples were kept at −20 °C for further analyzes after 24 h. In addition, thermal pasteurization (90 °C, 1 min) was also applied to compare the results with those obtained from HPP treatment.

### 2.3 In Vitro Digestion Model

The standardized static in vitro digestion method^21^ was adapted with some modifications to make it suitable for liquid foods. Since the digestion of liquid foods in the mouth is reported to be minimal, the method was modified to start with gastric digestion. Gastric and intestinal fluids are prepared as specified in the protocol.

To simulate gastric digestion, 10 mL of SCJ samples were combined with 7.5 mL of gastric fluid, 1.6 mL of pepsin solution (25,000 U/mL), and 5 μL of 0.3 M calcium chloride. The pH was adjusted to 3.0 using 1 M hydrochloric acid, and additional distilled water was added to reach a total volume of 20 mL. The mixture was then shaken at 90 rpm in water bath (Memmert SV 1422, Nürnberg, Germany) at 37 °C for 2 h. Following the simulation of gastric digestion step, 5 mL aliquots were collected from each sample.

To simulate intestinal digestion, the remaining mixture was combined with 8.25 mL of duodenal fluid, 3.75 mL of pancreatin (800 U/mL), 1.875 mL of bile (160 mM), and 30 μL of 0.3 M calcium chloride. The pH of the mixture was set to 7.0 using 1 M sodium hydroxide. Subsequently, the total volume was adjusted to 30 mL using distilled water, and the mixture was shaken at 90 rpm in water bath at 37 °C for 2 h. After the simulation of intestinal digestion, 5 mL aliquots were collected from each sample.

Blank samples (containing the same amount of water instead of SCJ) were also incubated under the same conditions described above and utilized to correct for interferences from the digestive fluids.

Samples obtained from the simulated gastric and intestinal digestion phases were centrifuged (Hettich Zentrifugen Universal 32R, Tuttlingen, Germany) at 23,000*g* for 5 min at 4 °C, and the resulting supernatants were stored at −20 °C for subsequent analysis.

### 2.4 Quantification of Total Phenolics

The total phenolic content of the samples was measured using the Folin-Ciocalteau method described by Spanos and Wrolstad^22^ with some modifications. 100 μL of sample was mixed with 900 μL of distilled water in a glass test tube, followed by the addition of 0.75 mL of 0.2 N Folin-Ciocalteau reagent. After a 5 min incubation period, 0.75 mL of saturated sodium carbonate solution was added to the mixture. The test tubes were then left to stand at room temperature in the dark for 90 min. Subsequently, the absorbance was measured at 765 nm,^22^ and the results were presented in terms of mg gallic acid equivalent (GAE) per 100 mL of sample.

### 2.5 Quantification of Total Flavonoids

The total flavonoid content of the samples was measured using the colorimetric method described by Dewanto et al.^23^ with some modifications. 0.25 mL of sample was mixed with 1.25 mL of distilled water in a glass test tube, followed by the addition of 75 μL of 5% sodium nitrite solution. After a 6 min incubation period, 150 μL of 10% aluminum chloride hexahydrate solution was added to the mixture. The test tubes were incubated for another 5 min, followed by the addition of 0.5 mL of 1 M sodium hydroxide solution. The volume was then adjusted to 2.5 mL with distilled water. Subsequently, the test tubes were vortexed and the absorbance was measured at 510 nm.^23^ The results were presented as mg catechin equivalent (CE) per 100 mL of sample.

### 2.6 Quantification of Total Anthocyanins

The total monomeric anthocyanin content of the samples was measured using the AOAC 2005.02 official pH differential method.^24^ Absorbance was measured at 520 and 700 nm in buffers at pH 1.0 (0.025 M potassium chloride) and pH 4.5 (0.4 M sodium acetate) and calculated using the following equation

$$total\;anthocyanin\;content\;\left(cyanidin3‐O‐glucoside\;equivalents,\;mg/L\right)=\frac{A\times MW\times DF\times 10^{3}}{\varepsilon \times l}$$

where *A* = (*A*_520 nm_ – A_700 nm_)_pH 1.0_ – (A_520 nm_ – A_700 nm_)_pH 4.5_, MW molecular weight of cyanidin 3-*O*-glucoside (449.2 g/mol), DF dilution factor, 10^3^ factor for conversion from g to mg, ε molar extinction coefficient of cyanidin 3-*O*-glucoside (26,900 L/(mol·cm)), and *l* path length (cm). The results were presented as mg cyanidin 3-*O*-glucoside equivalent (C3GE) per L of sample.

### 2.7 Quantification of Total Antioxidant Capacity

CUPRAC assay was conducted according to the method described by Apak et al.,^25^ with some modifications. In a glass test tube, 100 μL of sample was mixed with 1 mL of copper­(II) chloride, followed by 1 mL of neocuproine solution, 1 mL of ammonium acetate buffer, and finally 1 mL of distilled water, in the given order. The mixture was then vortexed and left to stand at room temperature in the dark for 30 min. Subsequently, the absorbance was measured at 450 nm, and the results were presented as mg Trolox equivalent (TE) per 100 mL of sample.

### 2.8 Quantification of Individual Polyphenolic Compounds

The individual polyphenolic compounds were identified and quantified using a HPLC system (Waters 2695) coupled to a PDA detector (Waters 2996), as previously described.^26^ Chromatographic separation was accomplished using a C18 column (250 × 4.6 mm, 5 μm; Supelcosil, Sigma-Aldrich) at a temperature of 40 °C. Samples, consisting of 10 μL extracts filtered through 0.45 μm membrane filters, were introduced into the system using mobile phases A (TFA/MQ water, 1:1000, v/v) and B (TFA/acetonitrile, 1:1000, v/v) at a flow rate of 1 mL/min. The gradient elution profile was as the following: starting with 5% B at 0 min, increasing to 35% B over 0–45 min, further to 75% B over 45–47 min, returning to 35% B over 47–49 min, and finally reverting to initial conditions at 50 min. Spectral measurements were recorded at 280, 312, 360, and 520 nm. For quantification purposes, epicatechin, cyanidin 3-*O*-glucoside, chlorogenic acid, quercetin 3-*O*-glucoside, rutin and kaempferol standards were employed, showing good linearity (*R* ^2^ ≥ 0.99) within the specified range (0.1–200 ppm). Results were presented as mg/100 mL of sample.

### 2.9 Statistical Analysis

All analyzes were conducted with three replicates. Statistical analysis was performed using SPSS software (version 29.0, SPSS Inc., Chicago, IL, USA). Different treatments were compared using one-way analysis of variance (ANOVA), followed by a Tukey post hoc test with a significance level of *p* < 0.05.

## 3 Results and Discussion

### 3.1 Total Phenolic Content (TPC)

As shown in Table 1, both thermal pasteurization and HPP treatments significantly reduced the TPC of undigested SCJ (6–52%) (*p* < 0.05), except for the HPP treatment carried out at 500 MPa for 20 min. These results are in agreement with a study that also showed a significant reduction in TPC in apples (14–47%) as a result of HPP treatment.^27^ It has been suggested that the reduction of TPC after HPP treatment might be associated with the residual activity of polyphenoloxidase (PPO).^28^ On the other hand, several other studies on fruit juices indicated that HPP treatment does not significantly affect the TPC,^29,30^ similar to our results obtained after treatment of SCJ at 500 MPa for 20 min. This suggests that the outcome may depend on the specific process conditions. Increasing the HPP treatment time at a constant pressure significantly enhanced the retention of TPC (*p* < 0.05). Moreover, all SCJ that received HPP treatment at varying pressures for 20 min had significantly higher TPC compared to those subjected to thermal pasteurization (*p* < 0.05). Other studies on strawberry^31^ and açaí^29^ juices also highlighted the advantage of HPP treatment over thermal pasteurization in terms of TPC retention. Similarly, consistent with findings reported in the literature,^32^ increasing the pressure during HPP treatment also enhanced the retention of TPC.

**Table 1:** Changes in Total Phenolic Content of Sour Cherry Juice Subjected to HPP and Thermal Pasteurization Treatments During Simulated In Vitro Digestion (mg GAE/100 mL)^a^

treatment	undigested	gastric digestion	intestinal digestion
control	788.3 ± 7.8 a,A	571.7 ± 18.2 b,B	443.3 ± 24.5 cd,C
thermal pasteurization	542.6 ± 12.5 d,A	506.7 ± 2.9 cd,AB	468.6 ± 24.5 bc,B
HPP/5 min			
300 MPa	377.8 ± 10.3 g,B	461.0 ± 12.2,A	402.7 ± 28.6 de,B
400 MPa	434.3 ± 14.2 f,A	373.8 ± 6.7 f,B	329.2 ± 8.6 f,C
500 MPa	455.9 ± 10.2 ef,B	472.3 ± 7.8 cd,AB	480.9 ± 8.6 bc,A
HPP/10 min			
300 MPa	435.2 ± 5.3 f,A	409.1 ± 16.1 ef,A	349.0 ± 21.6 ef,B
400 MPa	451.7 ± 6.6 ef,A	494.4 ± 33.2 cd,A	441.4 ± 22.0 cd,A
500 MPa	485.8 ± 4.0 e,AB	478.9 ± 4.9 cd,B	524.3 ± 28.3 b,A
HPP/20 min			
300 MPa	660.9 ± 19.0 c,A	459.6 ± 4.5 de,B	464.9 ± 4.3 bcd,B
400 MPa	741.9 ± 29.2 b,A	526.5 ± 10.7 bc,B	517.7 ± 4.3 b,B
500 MPa	793.0 ± 28.0 a,B	844.0 ± 44.6 a,B	965.3 ± 33.0 a,A

a Results are expressed as mean ± standard deviation. Different lower-case letters within the columns and upper-case letters within the rows represent statistically significant differences (*p* < 0.05).

Although the results obtained after the gastric digestion phase varied compared to the undigested phase, TPC was better preserved in HPP-treated SCJ at higher pressure (500 MPa) (104–109%) compared to both untreated (73%) and thermally pasteurized (93%) samples. During the intestinal digestion phase, the HPP-treated SCJ samples retained higher TPC values (85–114%) than the untreated SCJ (78%), compared to the gastric digestion phase. In particular, HPP treatments applied at 500 MPa showed significantly higher retention compared to those applied at lower pressures. It has been reported that HPP might release nonextractable phenolics at increased pressures,^33^ which may explain the findings in the current study. Both thermal pasteurization (86%) and HPP treatment (70–122%) increased the bioaccessibility of total phenolics in SCJ compared to untreated samples (56%). A previous study with mandarin juice^34^ also reported increased bioaccessibility of TPC in HPP-treated samples compared to untreated samples. HPP applied to food materials can generate a large pressure difference across the cell membrane, resulting in enhanced cell permeability, mass transfer, and the release of matrix-bound phenolic compounds.^35^ Nevertheless, the TPC assay using the Folin–Ciocalteu reagent is not specific to phenolic compounds. Reducing agents like ascorbic acid, citric acid or simple sugars, can interfere with the analysis, potentially leading to an overestimation of TPC.^36^ To address this limitation, we supplemented these findings by identifying individual polyphenolic compounds using HPLC-PDA.

### 3.2 Total Flavonoid Content (TFC)

In general, the changes in TFC of SCJ subjected to HPP and thermal pasteurization exhibited a trend similar to the results observed for TPC. Both thermal pasteurization and HPP treatments significantly reduced the TFC of undigested SCJ (12–39%) (*p* < 0.05), except for the HPP treatments carried out at 500 MPa for 10 and 20 min (Table 2). These findings align with a study that reported a reduction in TFC in durian fruit puree (10%) after HPP treatment.^37^ Similarly, our results for SCJ treated at 500 MPa for 10 and 20 min, no significant differences were observed in between HPP-treated and untreated carrot juice.^38^ Extending the HPP treatment duration from 5 min to 10 or 20 min at a constant pressure enhanced the TFC of SCJ (*p* < 0.05), while no statistically significant difference was observed between the treatments applied for 10 and 20 min. Moreover, increasing the pressure of HPP treatment at a constant duration improved the retention of TFC. Indeed, SCJ treated with HPP at 500 MPa for 10 and 20 min showed significantly higher retention of TFC (96–97%) compared to SCJ subjected to thermal pasteurization (73%). A previous study also found that HPP-treated pineapple juice retained a higher TFC compared to thermally treated juice.^39^

**Table 2:** Changes in Total Flavonoid Content of Sour Cherry Juice Subjected to HPP and Thermal Pasteurization Treatments during Simulated In Vitro Digestion (mg CE/100 mL)^a^

treatment	undigested	gastric digestion	intestinal digestion
control	755.1 ± 3.2 a,A	362.1 ± 11.1 abc,C	449.1 ± 9.7 a,B
thermal pasteurization	554.4 ± 11.6 cde,A	343.9 ± 17.0 bcd,B	244.2 ± 37.1 e,C
HPP/5 min			
300 MPa	447.7 ± 13.7 f,A	298.9 ± 29.5 d,B	324.6 ± 4.9 cd,B
400 MPa	501.8 ± 17.0 ef,A	337.9 ± 16.5 bcd,B	364.9 ± 17.5 cd,B
500 MPa	622.5 ± 73.1 bcd,A	374.7 ± 4.2 abc,B	435.1 ± 12.9 a,B
HPP/10 min			
300 MPa	532.6 ± 9.6 def,A	322.5 ± 17.5 cd,B	328.4 ± 8.4 cd,B
400 MPa	639.3 ± 22.3 bc,A	332.6 ± 11.1 bcd,C	372.6 ± 4.6 bc,B
500 MPa	703.7 ± 42.6 ab,A	374.0 ± 8.4 abc,C	466.0 ± 31.9 a,B
HPP/20 min			
300 MPa	531.9 ± 30.8 def,A	369.5 ± 11.1 abc,B	314.4 ± 4.9 d,C
400 MPa	640.0 ± 20.7 bc,A	387.4 ± 27.6 ab,B	355.1 ± 14.3 cd,B
500 MPa	696.8 ± 51.0 ab,A	414.0 ± 33.8 a,B	421.4 ± 13.3 ab,B

a Results are expressed as mean ± standard deviation. Different lower-case letters within the columns and upper-case letters within the rows represent statistically significant differences (*p* < 0.05).

Gastric digestion significantly reduced the TFC of all SCJ samples (28–53%) (*p* < 0.05). Although there was no consistent trend in TFC changes during the intestinal digestion phase compared to the gastric digestion phase, we observed that increasing the pressure of HPP treatment at a constant duration resulted in higher TFC retention. In contrast, increasing the application time at a constant pressure did not lead to a significant change (*p* > 0.05). Overall, the TFC bioaccessibility of all HPP-treated SCJ samples (60–82%) was higher than that of thermally pasteurized SCJ (51%). Additionally, all HPP-treated SCJ samples at 500 MPa (74–82%) demonstrated greater TFC bioaccessibility compared to untreated samples (61%). This finding corroborates our results with TPC and aligns with a previous study on mandarin juice,^34^ which also reported higher bioaccessibility of TFC in HPP-treated samples compared to untreated ones. Nonetheless, the assay used to determine TFC is not solely specific to flavonoids. In addition to flavonoids, phenolic acids also produce a fairly strong reaction in the assay, whereas most flavonoids, except for flavan-3-ols, yield poor reactions in this assay.^40^ Given the lack of specificity in this assay, in the current study, we also conducted HPLC-PDA analysis of flavonoids to attain more accurate results.

### 3.3 Total Monomeric Anthocyanin Content (TMAC)

In contrast to the results for TPC and TFC, TMAC of SCJ treated with HPP and thermal pasteurization did not differ significantly from the control (*p* > 0.05) (Table 3). Similarly, other studies on the effect of HPP on total anthocyanin retention in blueberries^41^ and strawberries^42^ have also reported negligible changes. The sugar-rich matrix of SCJ may provide a protective environment for anthocyanins, protecting them from degradation.^43^ Furthermore, anthocyanins are pH-sensitive and tend to form stable structures in acidic conditions. Given the acidic nature of SCJ, this characteristic likely contributed to the stability of anthocyanins during processing.^44^ Similarly, extending the HPP treatment duration or increasing the applied pressure showed no statistically significant effect on TMAC (*p* > 0.05). Another study investigating the effect of HPP on strawberry puree also observed an insignificant influence of pressure and time on TMAC.^45^

**Table 3:** Changes in Total Monomeric Anthocyanin Content of Sour Cherry Juice Subjected to HPP and Thermal Pasteurization Treatments during Simulated In Vitro Digestion (mg C3GE/L)^a^

treatment	undigested	gastric digestion	intestinal digestion
control	220.8 ± 13.8 ab,A	150.2 ± 4.0 abc,B	76.8 ± 1.3 f,C
thermal pasteurization	193.6 ± 12.7 b,A	165.0 ± 5.2 ab,B	159.4 ± 18.3 ab,B
HPP/5 min			
300 MPa	218.3 ± 4.9 ab,A	104.5 ± 1.9 d,B	49.2 ± 6.9 g,C
400 MPa	225.1 ± 3.7 ab,A	134.9 ± 11.9 bcd,B	108.8 ± 7.5 de,C
500 MPa	237.0 ± 16.5 ab,A	165.3 ± 15.6 ab,B	123.9 ± 1.3 cd,C
HPP/10 min			
300 MPa	220.8 ± 8.2 ab,A	123.1 ± 14.2 cd,B	83.0 ± 4.0 f,C
400 MPa	229.1 ± 9.8 ab,A	135.1 ± 20.8 bcd,B	138.7 ± 5.3 bc,B
500 MPa	241.3 ± 13.5 a,A	174.7 ± 19.3 a,B	152.3 ± 7.6 ab,B
HPP/20 min			
300 MPa	247.6 ± 13.1 a,A	114.7 ± 6.1 d,B	94.7 ± 1.0 ef,B
400 MPa	254.5 ± 22.1 a,A	134.9 ± 3.0 bcd,B	150.1 ± 6.4 ab,B
500 MPa	261.8 ± 31.4 a,A	175.0 ± 3.1 a,B	164.8 ± 7.0 a,B

a Results are expressed as mean ± standard deviation. Different lower-case letters within the columns and upper-case letters within the rows represent statistically significant differences (*p* < 0.05).

Similar to the results observed for TFC, gastric digestion significantly reduced the TMAC in all SCJ samples (15–54%) (*p* < 0.05). During the intestinal digestion phase, TMAC decreased further in most samples. However, increasing the pressure and duration of HPP treatment yielded TMAC levels comparable to those observed after the gastric digestion phase. The TMAC bioaccessibility of HPP-treated samples (38–63%) was higher than that of the control (35%), except for the treatment conducted at 300 MPa for 5 min (23%). In contrast, thermally treated SCJ exhibited the highest bioaccessible TMAC (82%). This observation may be explained by the partial inactivation of PPO during HPP. While thermal pasteurization typically results in full enzyme inactivation, HPP at 500 MPa may not completely inactivate PPO, leading to continued enzymatic degradation of anthocyanins during digestion. Previous studies have shown that moderate pressures may retain PPO activity, whereas pressures ≥ 600 MPa are required for full inactivation.^46^ For example, de Oliveira et al.^47^ reported significant anthocyanin losses in jussara juice treated with HPP at 500 MPa, attributing this to incomplete PPO inactivation. Therefore, despite the high pressure and long duration, residual enzymatic activity may still limit anthocyanin bioaccessibility under HPP.

### 3.4 Total Antioxidant Capacity (TAC)

In this study, CUPRAC assay was utilized to evaluate changes in TAC following HPP treatment at varying pressures and durations. CUPRAC method is notable for its ability to measure both lipophilic and hydrophilic antioxidants. Key advantages of this method include its operation at physiological pH, the capacity to generate a linear response across a wide concentration range, and high reproducibility.^36^ Thermal treatment significantly reduced the TAC of SCJ samples (27%), whereas HPP treatment preserved TAC levels comparable to the control (*p* > 0.05) (Table 4). The detrimental effect of thermal treatment is likely due to the degradation of heat-sensitive antioxidants, such as vitamin C. Extending the HPP treatment duration or increasing the applied pressure generally had no statistically significant effect on TAC (*p* > 0.05). Similarly, a previous study on the retention of TAC in cranberrybush purée following HPP also found no significant changes with extended application time or increased pressure.^48^

**Table 4:** Changes in Total Antioxidant Capacity of Sour Cherry Juice Subjected to HPP and Thermal Pasteurization Treatments during Simulated In Vitro Digestion (mg TE/100 mL)^a^

treatment	undigested	gastric digestion	intestinal digestion
control	475.8 ± 8.7 ab,A	325.1 ± 19.7 ab,C	395.0 ± 24.6 ab,B
thermal pasteurization	349.3 ± 18.2 c,B	286.7 ± 11.1 abc,C	418.6 ± 24.5 a,A
HPP/5 min			
300 MPa	426.7 ± 31.1 b,A	264.1 ± 3.1 bc,B	256.3 ± 4.9 e,B
400 MPa	426.7 ± 25.7 b,A	290.0 ± 1.7 abc,B	274.9 ± 11.7 de,B
500 MPa	434.6 ± 21.7 b,A	272.5 ± 29.5 abc,B	283.4 ± 3.2 de,B
HPP/10 min			
300 MPa	444.1 ± 12.8 ab,A	317.7 ± 28.1 ab,B	315.0 ± 14.6 cd,B
400 MPa	449.7 ± 17.4 ab,A	248.7 ± 11.6 c,C	349.7 ± 40.1 bc,B
500 MPa	465.2 ± 30.5 ab,A	274.9 ± 18.6 abc,B	315.0 ± 20.7 cd,B
HPP/20 min			
300 MPa	440.3 ± 7.7 b,A	329.8 ± 26.0 a,B	298.2 ± 21.1 cde,B
400 MPa	458.8 ± 12.6 ab,A	329.1 ± 40.9 a,B	308.0 ± 9.4 cde,B
500 MPa	497.4 ± 7.8 a,A	322.4 ± 11.3 ab,C	439.1 ± 9.9 a,B

a Results are expressed as mean ± standard deviation. Different lower-case letters within the columns and upper-case letters within the rows represent statistically significant differences (*p* < 0.05).

As observed with TFC and TMAC, gastric digestion significantly decreased the TAC in all SCJ samples (18–45%) (*p* < 0.05). During the intestinal digestion phase, TAC levels increased in the control and thermally treated samples compared to those observed during gastric digestion. In contrast, most HPP-treated samples exhibited no statistically significant change. HPP treatment generally reduced the bioaccessibility of TAC (60–78%) compared to the control (83%), except for the sample processed at 500 MPa for 20 min (88%). The significantly higher TAC values observed in samples treated at 500 MPa for 20 min may be attributed to enhanced extractability of antioxidant compounds under high pressure.^29^ Consistent with the findings for TMAC, thermally treated SCJ demonstrated the highest bioaccessible TAC (120%). This observation may be attributed to the fact that thermal degradation products of phenolic compounds can retain strong antioxidant capacity.^29^

### 3.5 Individual Polyphenolic Compounds

In this study, individual phenolic compounds were analyzed in samples subjected to low, midrange, and high HPP parameters. Six major polyphenols were identified in SCJ samples, comprising five flavonoids—epicatechin, cyanidin 3-*O*-glucoside, quercetin 3-*O*-glucoside, rutin, and kaempferol—and one phenolic acid, chlorogenic acid (Table 5). Epicatechin was determined as the predominant compound in all SCJ samples, accounting for 54–62% of the total polyphenols identified. Although some differences were observed in individual polyphenolic compounds, thermal treatment overall led to a statistically significant reduction in total polyphenolic compound level (43%) (*p* < 0.05). While the effect of HPP was less pronounced compared to thermal treatment, it still caused a reduction in the total polyphenolic content when applied under low to moderate pressure and duration (27–35%). On the other hand, although statistically not significant, high pressure and long duration (500 MPa for 20 min) increased the levels of total polyphenolics (11%). These results align with studies on Italian apples^27^ and pomegranate juice,^18^ where epicatechin and cyanidin 3-*O*-glucoside levels, respectively, were found to increase with higher pressure.

**Table 5:** Changes in Individual Polyphenolic Compounds of Sour Cherry Juice Subjected to HPP and Thermal Pasteurization Treatments during Simulated In Vitro Digestion (mg/100 mL)^a^

treatment	undigested	gastric digestion	intestinal digestion
Epicatechin			
control	756.2 ± 65.1 ab,A	143.2 ± 34.2 bc,B	171.5 ± 52.8 b,B
thermal pasteurization	451.8 ± 114.1 c,A	340.0 ± 0.1 a,A	340.4 ± 33.4 a,A
HPP/300 MPa, 5 min	472.1 ± 66.9 c,A	224.9 ± 45.0 b,B	331.9 ± 8.3 a,B
HPP/400 MPa, 10 min	547.2 ± 38.2 bc,A	315.4 ± 46.3 a,B	281.2 ± 1.2 a,B
HPP/500 MPa, 20 min	948.4 ± 84.7 a,A	97.9 ± 3.9 c,B	126.5 ± 0.5 b,B
Chlorogenic acid			
control	288.2 ± 99.8 a,A	180.0 ± 1.2 b,A	198.2 ± 0.9 a,A
thermal pasteurization	156.5 ± 4.7 a,B	357.1 ± 26.5 a,A	154.1 ± 12.0 b,B
HPP/300 MPa, 5 min	193.3 ± 3.0 a,A	131.2 ± 13.7 c,B	187.1 ± 8.7 a,A
HPP/400 MPa, 10 min	226.2 ± 32.3 a,A	197.8 ± 5.2 b,A	85.1 ± 0.1 c,B
HPP/500 MPa, 20 min	246.9 ± 46.5 a,A	140.0 ± 1.9 c,B	182.8 ± 0.9 a,AB
Cyanidin 3-*O*-glucoside			
control	230.2 ± 83.5 a,A	160.9 ± 5.2 a,A	118.8 ± 2.8 b,A
thermal pasteurization	118.6 ± 1.2 a,A	165.1 ± 45.3 a,A	128.8 ± 12.4 b,A
HPP/300 MPa, 5 min	146.3 ± 9.5 a,A	111.4 ± 6.5 a,B	150.8 ± 3.2 a,A
HPP/400 MPa, 10 min	157.9 ± 3.1 a,A	126.7 ± 3.2 a,B	100.7 ± 0.8 c,C
HPP/500 MPa, 20 min	233.8 ± 70.7 a,A	139.4 ± 16.1 a,A	144.9 ± 0.1 a,A
Quercetin 3-*O*-glucoside			
control	62.8 ± 20.9 a,A	38.1 ± 0.1 b,AB	28.9 ± 0.6 cd,B
thermal pasteurization	32.7 ± 0.1 a,B	74.5 ± 7.1 a,A	30.7 ± 2.2 bc,B
HPP/300 MPa, 5 min	40.8 ± 0.9 a,A	30.1 ± 4.3 b,B	38.2 ± 1.1 a,A
HPP/400 MPa, 10 min	43.1 ± 0.7 a,A	29.2 ± 0.7 b,B	24.4 ± 3.1 d,C
HPP/500 MPa, 20 min	63.8 ± 19.2 a,A	35.9 ± 1.3 b,AB	35.2 ± 2.7 ab,B
Rutin			
control	23.1 ± 9.1 a,A	15.8 ± 2.4 b,A	11.4 ± 1.4 ab,A
thermal pasteurization	12.0 ± 0.5 a,B	28.3 ± 2.6 a,A	12.4 ± 2.2 ab,B
HPP/300 MPa, 5 min	16.0 ± 0.6 a,A	11.0 ± 0.8 b,C	13.6 ± 0.1 a,B
HPP/400 MPa, 10 min	15.1 ± 0.2 a,A	10.6 ± 0.1 b,B	9.4 ± 1.1 b,B
HPP/500 MPa, 20 min	24.3 ± 7.8 a,AB	28.3 ± 5.1 a,A	13.0 ± 0.2 a,B
Kaempferol			
control	3.7 ± 0.2 a,A	2.6 ± 0.7 b,B	1.4 ± 0.1 c,C
thermal pasteurization	3.0 ± 0.2 b,B	6.5 ± 0.7 a,A	1.8 ± 0.2 bc,C
HPP/300 MPa, 5 min	4.0 ± 0.1 a,A	2.3 ± 0.1 b,C	3.7 ± 0.1 a,B
HPP/400 MPa, 10 min	3.9 ± 0.2 a,A	2.4 ± 0.1 b,B	1.9 ± 0.3 bc,B
HPP/500 MPa, 20 min	3.9 ± 0.4 a,A	1.8 ± 0.1 b,B	2.4 ± 0.7 b,B
TOTAL			
control	1364.3 ± 148.3 ab,A	540.7 ± 29.5 c,B	530.2 ± 47.1 b,B
thermal pasteurization	774.6 ± 119.9 c,B	971.5 ± 8.5 a,A	668.2 ± 62.0 a,B
HPP/300 MPa, 5 min	881.8 ± 49.2 c,A	511.0 ± 68.7 c,C	725.4 ± 21.3 a,B
HPP/400 MPa, 10 min	993.3 ± 74.7 bc,A	682.0 ± 55.6 b,B	502.7 ± 4.0 b,C
HPP/500 MPa, 20 min	1520.7 ± 229.7 a,A	443.3 ± 7.8 c,B	504.9 ± 3.8 b,B

a Results are expressed as mean ± standard deviation. Different lower-case letters within the columns and upper-case letters within the rows represent statistically significant differences (*p* < 0.05).

Gastric digestion decreased the total polyphenolics in both the control and HPP-treated samples (31–71%) (*p* < 0.05), whereas it significantly increased the total polyphenolic content in the thermally treated samples (25%) (*p* < 0.05). Although varying trends were observed following intestinal digestion, low- and midrange HPP treatments were found to enhance the bioaccessibility of total polyphenolics (51–82%) compared to the control (39%). Increase in the applied pressure and duration was found to reduce the bioaccessibility of polyphenolic compounds. The reduction in the bioaccessibility of polyphenolic compounds in SCJ may result from structural modifications, such as polymerization or degradation of polyphenols, induced by increased pressure and duration. Additionally, HPP can enhance interactions between polyphenols and other juice components, forming insoluble complexes or trapping polyphenols within the juice matrix, reducing their release during digestion. Oxidative changes under pressure may also contribute to the formation of less bioaccessible derivatives. In line with the results for TMAC and TAC, thermally treated SCJ exhibited the highest bioaccessible total polyphenolic compound content (86%). The higher bioaccessible polyphenolic content in thermally treated SCJ compared to control and HPP-treated SCJ may be attributed to the thermal breakdown of cell walls and matrix components, which enhances the release of bound polyphenols. Additionally, heat treatment can inactivate polyphenol-degrading enzymes and hence promote the bioaccessibility of polyphenols.

## 4 Conclusions

This study aimed to evaluate, for the first time, the effects of HPP treatment at different pressures and durations on the content and in vitro bioaccessibility of TPC, TFC, TMAC, TAC, and individual polyphenolic compounds in SCJ. The findings showed that both thermal pasteurization and HPP significantly improved the bioaccessibility of polyphenols compared to untreated samples, with thermal treatment achieving the highest levels for TMAC, TAC, and total individual polyphenolics. HPP-treated samples demonstrated greater TFC bioaccessibility than thermally pasteurized SCJ, with treatment at 500 MPa notably enhancing bioaccessibility across most phenolic fractions due to increased cell permeability and mass transfer. However, HPP generally resulted in lower TAC bioaccessibility compared to the control, except for samples treated at 500 MPa for 20 min, highlighting the complicated relationship between processing parameters and phenolic compound stability. Future studies should investigate the effects of HPP on the enzymatic activity of SCJ, focusing on key enzymes such as PPO, peroxidase, and β-glucosidase. Examining the stability and bioaccessibility of polyphenolic compounds in SCJ under different storage conditions could offer valuable insights into their long-term behavior. Furthermore, conducting sensory evaluations of HPP-treated juice over time would help assess consumer acceptance and refine processing parameters to preserve both nutritional value and sensory quality. While HPP offers superior preservation of nutritional quality and fresh-like characteristics in fruit and vegetable products, it necessitates a significantly higher capital investment and operational cost compared to traditional thermal pasteurization. However, the benefits of HPP in preserving bioactive compounds and enhancing product quality may justify the higher costs, particularly in the functional beverage markets.
